# Study on Microstructure and Thermal Cracking Sensitivity of Deposited Ti6Al4V/Inconel 718 Composites Made by Two-Wire Arc Additive Manufacturing by Current

**DOI:** 10.3390/ma17235989

**Published:** 2024-12-06

**Authors:** Peng Xia, Xin Ye, Guangshun Zhang

**Affiliations:** School of Materials Science and Engineering, Shanghai University of Engineering Science, Shanghai 201620, China; xia159112@163.com (P.X.); zhang16140225@163.com (G.Z.)

**Keywords:** arc additive manufacturing, microstructure, Ti6Al4V/Inconel 718 composites, mechanical properties

## Abstract

Ti6Al4V/Inconel 718 composites were prepared using arc additive manufacturing technology at different deposition currents. The properties of the composites directly influence the performance of the gradient materials, while heat input further affects the composites’ properties. The results indicate that at a deposition current of 35 A, Ti elements diffuse into the Inconel 718 alloy. Increasing the current leads to the formation of brittle intermetallic compounds such as TiNi, Cr_2_Ti, and Fe_2_Ti in the deposited layer. At deposition currents below 50 A, no cracks appear, but cracks develop at a current of 50 A. Additionally, the microhardness of the deposited layer increases with higher deposition currents. Compared to the 35 A condition, microhardness rises by 31.51% at a current of 50 A. This research can expand the application field of the arc additive manufacturing of direct deposition Ti6Al4V/Inconel 718 composites.

## 1. Introduction

The ongoing advancement of the aerospace sector has led to an increased demand for more sophisticated spacecraft, particularly in the area of dissimilar metal connection structures. These structures must now meet the dual requirements of high performance and lightweight design [1,2,3]. Therefore, more and more researchers have begun to pay attention to the connection of dissimilar metals and obtained Ti/Al, Mg/Al, Fe/Al, Ti/Ni, and other composite components [4,5,6,7,8]. Among them, Ti6Al4V alloy and Inconel 718 alloy are widely used in the aerospace field due to their excellent properties, so there is increasing attention on the connection between titanium-based alloy and nickel-based superalloy [9,10]. The structure composed of the connection of Ti6Al4V alloy and Inconel 718 superalloy can effectively reduce the mass of the parts and adapt to high-temperature environments [11,12]. However, due to the differences in thermal physical property parameters such as the thermal expansion coefficient and thermal conductivity coefficient of dissimilar metals, cracks will occur in the connection process of titan-based alloy and nickel-based superalloy [13,14,15], and the preparation of gradient materials can improve this situation. In addition, when dissimilar metals are connected, composite materials are often formed at the joint, in which brittle intermetallic compounds (IMCs) such as Ti_2_Ni and Ni_3_Ti are easily formed, which further increases the brittleness of dissimilar joints [14,16,17]. Therefore, the microstructure and mechanical properties of composite materials play an important role in the preparation of gradient materials.

In the process of dissimilar metal connection, new composite materials will inevitably be formed, so some researchers achieve a dissimilar metal connection by changing the alloy’s composition layer by layer. Sun et al. [18] prepared the Inconel 625/Ti6Al4V component using laser additive manufacturing technology and studied the changes in the composition and structure of the alloy from Ti6Al4V to Inconel 625. The results show that the phase changes to α+β→α+β+Ti_2_Ni→Ti_2_Ni+β→Ti_2_Ni+Cr_2_Ni+γ-Ni, and a micro-crack occurs in the diffusion zone at 800 °C. Meng et al. [19] prepared Inconel 625/Ti6Al4V functionally graded materials utilizing laser synchronous preheating/non-preheating and found that preheating had a great influence on the formation of cracks and precipitates. Song et al. [10] used laser additive manufacturing technology to prepare composite materials with different Ti contents. The results showed that with the increase in the Ti content, the main phase of the prepared component changed to γ→Fe_2_Ti→TiNi. Onuike et al. [20] used laser additive manufacturing technology to prepare Inconel/Ti6Al4V gradient material and introduced a new phase Cr_3_C_2_ by introducing VC as the intermediate layer to avoid the generation of a brittle phase. Wang et al. [15] prepared the Ti6Al4V/Inconel718 component using linear laser additive manufacturing technology. The results showed that the phase microstructure of the Ti6Al4V/Inconel 718 component was γ+Laves→α+β+Ti_2_Ni+TiNi+Ni_3_Ti→α+β from the bottom to the top. However, when the welding wire is used as a raw material for laser additive manufacturing, there will be stratification. Most researchers prefer to use lasers as heat sources to prepare functionally graded materials. However, because the laser is used as the deposition heat source, it needs high power to melt the wire or powder, which greatly increases the deposition cost. When the arc is used as the deposition heat source, it is not necessary to use high power to melt the wire material, and arc additive manufacturing has the advantages of high product efficiency, a high material utilization rate, and low equipment cost [21]. Huang et al. [21] used arc additive manufacturing technology to prepare TiNi gradient materials by controlling the wire feed speeds of Ti6Al4V and Inconel 625. The results showed that the use of arc could make the phase distribution of Ti_2_Ni and TiNi uniform and improve the compression performance and surface wear performance of TiNi components. Lu et al. [22] used arc additive manufacturing technology to prepare the functional gradient of TA1/Inconel 625 and studied its microstructure and mechanical properties. However, only a few studies have preliminarily discussed the use of arc additive manufacturing technology to connect Inconel 718 and Ti6Al4V alloys. In summary, there are few studies on the preparation of Ti6Al4V/Inconel 718 gradient materials by arc additive manufacturing; in particular, the discussion on microstructure and mechanical properties is relatively limited.

The premise of preparing gradient materials is to prepare intact composite materials, so the microstructure and mechanical properties of composite materials often affect the properties of gradient materials. In the process of deposition, heat input will affect the microstructure and mechanical properties of composite materials. In this paper, Ti6Al4V and Inconel 718 wires are selected as Ti6Al4V and Inconel 718 welding equipment, and the wire feeding speed of the two wires is fixed to prepare Ti6Al4V/Inconel 718 composite materials with different deposition currents. The effects of different deposition currents on the microstructure and mechanical properties of Ti6Al4V/Inconel 718 composites were investigated. By comparing the morphologies of the intermetallic phases in the composites under different deposition currents, the causes of the crack sections were analyzed. In addition, the microhardness of the samples prepared by different deposition currents was evaluated. It can expand the application field of arc additive manufacturing direct deposition Ti6Al4V/Inconel 718 composites.

## 2. Experimental Details

**Figure 1:**
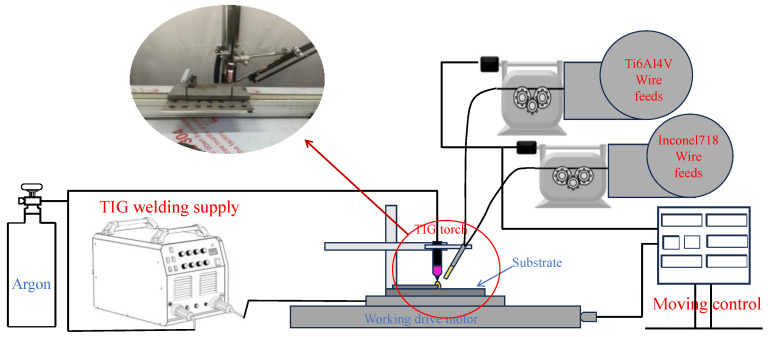
D-WAAM device diagram.

The experiment adopts a single arc deposition system, which mainly includes a TIG welding machine, servo motor mobile platform, and wire feeding mechanism. The working principle is shown in Figure 1. The Ti6Al4V and Inconel 718 wires are heated by an arc heat source. The two wires are placed on one side and the wire feeder can achieve different wire feed speeds. The system uses 99.99% high-purity argon as the protection gas.

The substrate was Inconel 718 and its dimensions were 100 mm × 50 mm × 4 mm. Before the experiment, the surface was polished with an angle grinder, the oxide layer was removed, and the surface was washed and dried with acetone and alcohol. A layer of Inconel 718 was deposited before the Ti6Al4V/Inconel 718 composite layer was deposited. Continued deposition occurred on the deposited Inconel 718 layer. The diameter of the Ti6Al4V and Inconel 718 wires is 1.2 mm, and the chemical composition of the two wires is shown in Table 1 and Table 2. The composition of the alloy is controlled by considering the wire feed speed ($V_{i}$), density, and diameter [23]. Table 3 shows the deposition parameters. The mass fraction ($W_{x}$) and atomic fraction ($A_{x}$) can be calculated using the following formula [24]:

(1) $$W_{x}=\frac{\sum V_{i}d_{i}\rho_{i}W_{x_{i}}}{\sum V_{i}d_{i}\rho_{i}}$$

(2) $$A_{x}=\frac{W_{x}/M_{x}}{\sum W_{x}/M_{x}}$$

where i is a metal wire (i = 1, 2, 1 means Ti6Al4V, 2 means Inconel 718), $W_{x_{i}}$ (x is an element in the Ti6Al4V and Inconel 718 alloys; i = 1, 2) is the mass fraction of an element in the wire, $V_{i}$(i = 1, 2) is the wire feeding speed of the wire, and the unit is mm/s. $d_{i}$(i = 1, 2) is the diameter of the wire in mm. $M_{x}$ (x is an element in the Ti6Al4V and Inconel 718 alloys) is the relative atomic mass and $\rho_{i}$(i = 1, 2) is the density of the wire, expressed in g/cm^3^.

The sample is cut along the longitudinal section, and the small sample is polished with 400–2000 purpose SiC sandpaper. Further polishing was carried out with a 1.5 particle size suspension. Finally, Kroll reagent (HF: HNO_3_: C_2_H_5_OH=7:2:1) was used for etching for about 10 S. The phase structure morphology between dissimilar metals was observed by a laser confocal microscope, and then the phase structure was analyzed by a high-magnification electron microscope. The microstructure of the samples was analyzed by scanning electron microscopy (SEM) and energy dispersive spectroscopy (EDS). The phase composition of the sample was analyzed by X-ray diffraction (XRD). The microhardness of the composite was measured by the Hv-1000 Vickers microhardness tester. The loading force was 200 g, and the residence time was 15 s.

## 3. Results and Discussion

### 3.1. Single-Layer Deposition Analysis

**Figure 2:**
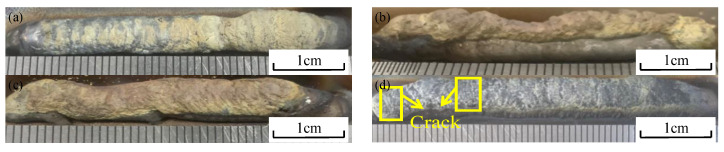
Sedimentary morphologies of different sedimentary currents: (**a**) 35 A, (**b**) 40 A, (**c**) 45 A, and (**d**) 50 A.

To determine the appropriate process parameters, the ratio of two types of welding wire is adjusted by varying the speed of the wire feeder. After fixing the wire feeding speed for both types of welding wire, different deposition currents are applied to prepare the sediment. Figure 2 shows the deposition morphologies under different deposition currents. As the current increases, the roughness of the deposited layer also increases significantly, primarily due to the increase in the viscosity of the liquid metal. Van et al. [25,26] studied the relationship between heat input and forming quality, and the results showed that the lower the heat input, the smoother the surface of the sedimentary components. This is consistent with the morphology of the samples deposited in this work. At higher cooling rates, the wettability and diffusivity of liquid metals are reduced, resulting in poorer adhesion. Furthermore, when the current reaches 50 A, cracks appear on the surface of the layer.

### 3.2. XRD Phase Analysis

**Figure 3:**
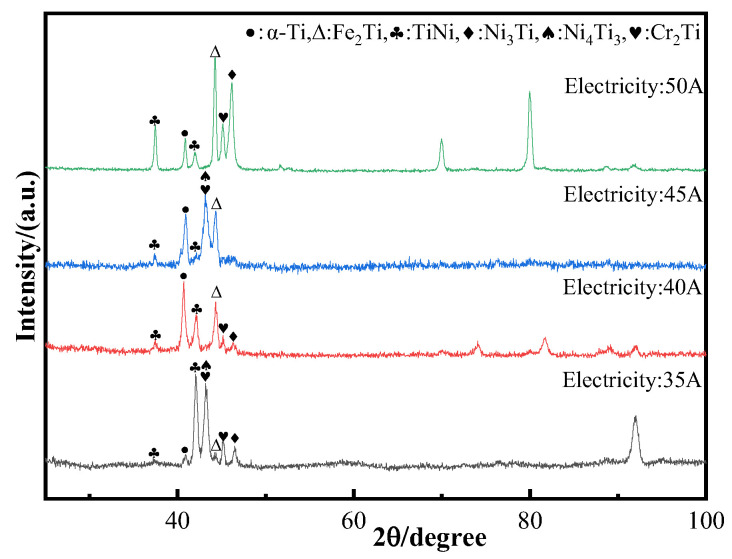
X-ray diffraction spectra of deposited products under different deposition currents.

The wire feed speed of Inconel 718 is twice that of Ti6Al4V. According to Formulas (1) and (2), when the element ratio of titanium to nickel is 1:2, a deposited layer is prepared using these two welding wires. During the deposition process, Ti-Ni intermetallic compounds are formed. In addition, Inconel 718 alloy also contains a certain amount of Cr and Fe, which may also form intermetallic compounds with Ti. The X-ray diffraction method was used to analyze the phases of the sediments prepared under different sedimentary currents, as shown in Figure 3. All the samples exhibited the TiNi phase, Ni_3_Ti phase, Cr_2_Ti phase, α-Ti phase, and Fe_2_Ti phase.

### 3.3. Organizational Characterization by Optical Microscopy

**Figure 4:**
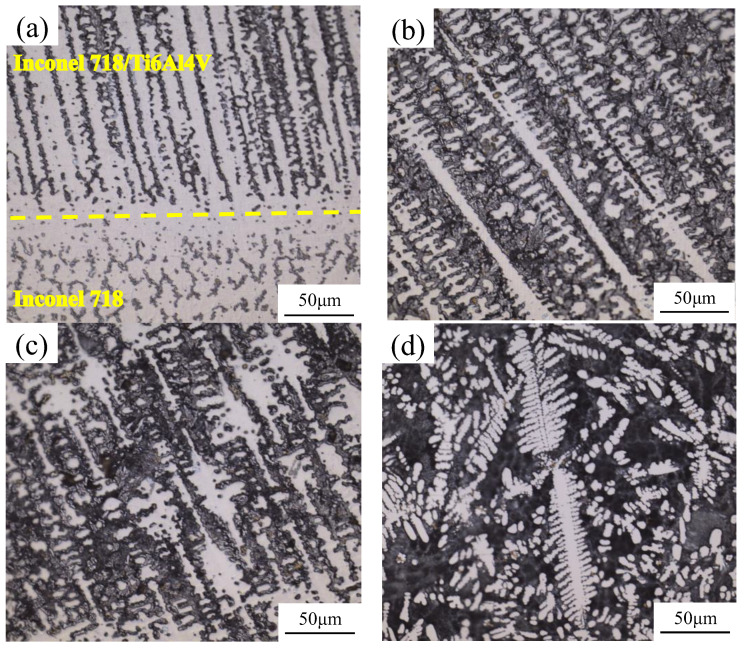
Optical microscope characterization of samples under different deposition currents: (**a**) 35 A, (**b**) 40 A, (**c**) 45 A, and (**d**) 50 A.

To study the effect of different heat inputs on the microstructure of the same composite material (V_Inconel 718_: V_Ti6Al4V_=2:1), the metallographic morphology of the samples was observed under different deposition currents using an optical microscope. Figure 4 displays the optical microscopy characterization for each deposition current. Figure 4a shows the optical microstructure at a deposition current of 35 A, with the Ti6Al4V/Inconel 718 composite in the upper part and the Inconel 718 layer in the lower part. The upper section is predominantly composed of columnar crystals, intermingled with some long black bars among the branches of the columnar crystals. In the WAAM process, the solid–liquid interface gradually advances into the liquid phase region. During this advancement, the solute undergoes redistribution, resulting in a phenomenon of component supercooling within a certain distance. This instability of the flat solid–liquid interface ultimately leads to the formation of columnar crystals. As the liquid phase region is influenced by solute redistribution, solute aggregation occurs, which is manifested as microscopic segregation [27]. From the Figure 4a, it can be observed that tiny secondary dendrites are present on the sides of the columnar crystals, with an average distance of 5.16 μm between these secondary dendrites.

With the increase in the deposition current, the columnar crystals in the deposited layer gradually undergo differentiation. Figure 4b shows the optical microstructure at a deposition current of 40 A. From the Figure 4b, it can be observed that some of the grains are arranged in an orderly manner, while a discontinuous black phase is present between the grains. In addition, long straight columnar crystals can be observed in the Figure 4b, which is formed due to the instability of the solid–liquid interface resulting from the supercooling of the composition. Under conditions of rapid cooling, columnar crystals are formed more readily. In comparison to Figure 4a, when the deposition current is 40 A, the heat input increases, leading to a longer temperature residence time on the workpiece under the same heat dissipation conditions. As a result, the cooling rate becomes relatively slower. At the microscopic level, the number of columnar crystals decreases, with some larger columnar crystals gradually transforming into smaller grains, leading to an increase in the spacing between columnar crystals. As some of the heat is conducted in the direction of the deposited additive, a negative temperature gradient develops in the liquid phase, perpendicular to the crystal axis. This results in the formation of a secondary crystal axis along the primary crystal axis [27]. The presence of secondary dendrites can be observed in the Figure 4b, with the average distance between the secondary dendrites measuring 6.38 μm.

When the deposition current is further increased, the metallographic morphology of the deposited layer is shown in Figure 4c. This Figure 4c illustrates the optical microstructure at a deposition current of 45 A, where fine columnar crystals and “leaf-like” grains can be observed. As the current increases, the grains grow further, with some secondary dendrites fusing during the growth process to form larger “leaf-like” grains that merge with the columnar crystal axis.

Figure 4d illustrates the optical microstructure at a deposition current of 50 A, where typical dendritic columnar crystals are observed, and the content of epitaxial crystals has increased. In comparison to the deposition current of 45 A, the increase in the heat input slightly extends the heat dissipation time, allowing for further grain growth and an increase in secondary dendrites. The average distance between the secondary dendrites is 3.96 μm. Additionally, a significant presence of grayish-black intra-crystalline structures can be observed in this region, which will necessitate further examination through EDS scanning analysis.

**Figure 5:**
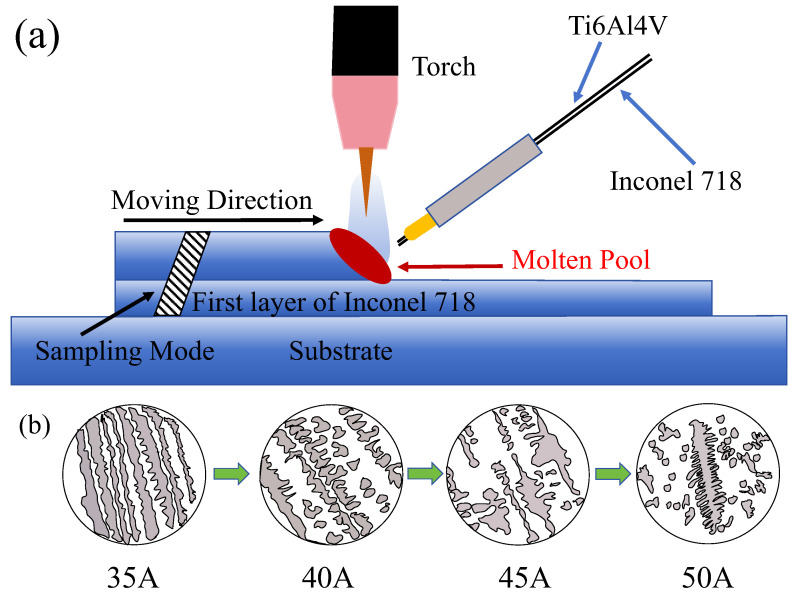
(**a**) Deposition diagram and (**b**) grain evolution diagram of the current 35 A–50 A deposition sample.

Figure 5 presents a schematic diagram illustrating the deposition process and the grain evolution of the sedimentary sample as the current increases. As the deposition current is gradually increased, the columnar crystals in the deposited samples evolve from a “long strip” morphology to a “rod-like” structure. With further increases in current, these “rod-like” dendrites begin to merge, resulting in the formation of “leaf-like” dendrites. When the current reaches 50 A, the “leaf” dendrites split into smaller dendrites. The sampling method for the sedimentary sample involves examining the cross-section of the additive body. When the deposition current is low (<50 A), the growth direction of the columnar crystals is perpendicular to the direction of the additive. However, as the deposition current reaches 50 A, the growth direction of a few columnar crystals remains perpendicular to the additive body, while most columnar crystals become parallel to the additive. This change may be attributed to the increased deposition current, which leads to an increase in heat input. In the Wire Arc Additive Manufacturing (WAAM) process, excessive heat diffuses along the formed additive, causing the growth direction of the columnar crystals to favor a parallel orientation with the additive body.

**Figure 6:**
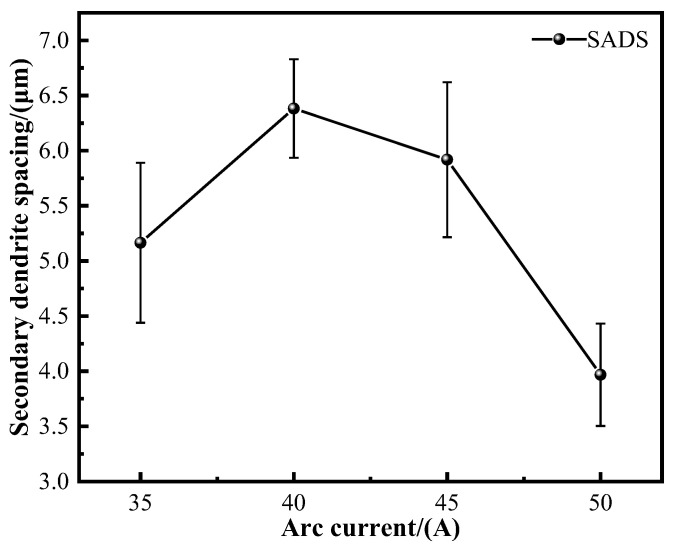
Statistical results of secondary dendrite spacing for each sedimentary current.

Figure 6 presents the statistical results of secondary dendrite arm spacing (SDAS) at different deposition currents. At deposition currents of 40 A and 45 A, the SDAS values are similar and larger than those at 35 A and 50 A. Notably, the minimum SDAS at 50 A is recorded at 3.96 μm. As the current increases, the heat input also rises, leading to an increase in the deposition temperature. This change enhances atomic mobility, increases the kinetic energy of certain atoms, and reduces the interfacial energy among grains, thereby promoting grain growth. The grain growth process is also clearly illustrated in Figure 5. When the deposition current is lower than 50 A, the change in secondary dendrite spacing SDAS is minimal, and all measured values are lower than those observed at 50 A. This phenomenon may be attributed to the limited heat input in the sedimentary layer and an excessively high cooling rate, which hinders the kinetic energy required for grain growth. Consequently, atomic migration is impeded, leading to a cessation of grain growth, and the secondary dendrite spacing does not exhibit significant changes. When the deposition current reaches 50 A, the increased heat input facilitates the further growth of some grains, while additional secondary dendrites also form, resulting in a decrease in secondary dendrite spacing. The morphology of the grains gradually shifts from columnar to dendritic, with the thickness of the columnar crystals also decreasing.

### 3.4. SEM and Element Content Analysis

**Figure 7:**
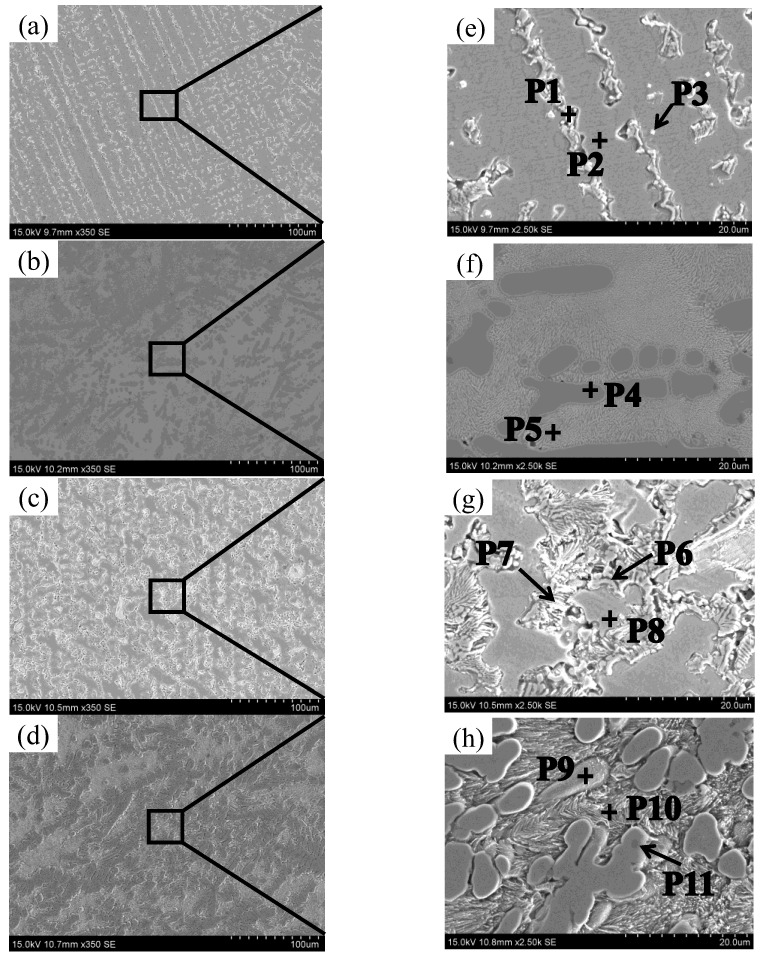
SEM scanning topography and EDS measuring point locations: (**a**) 30 A, (**b**) 40 A, (**c**) 45 A, (**d**) 50 A, (**e**) partial magnification in a, (**f**) partial magnification in b, (**g**) partial magnification in c, and (**h**) partial magnification in d.

To investigate the tissue composition of samples deposited under various current conditions, scanning electron microscopy (SEM) and energy dispersive spectrometry (EDS) point analyses were conducted on each deposited layer. The detailed results are presented in Figure 7 and Table 4. By combining the information from Figure 4a, Figure 7a,e, and Table 4, it can be concluded that the black phase (P1) between the columnar crystals exhibits the highest carbon (C) content, suggesting the presence of MC carbide. MC carbides play a crucial role in nickel-based alloys. Even a small amount of MC carbides can enhance strength by inhibiting dislocation movement and stabilizing grain boundaries [15]. At the position corresponding to the crystal axis (P2), the concentrations of Ni, Cr, and Fe are elevated. This may be attributed to the low deposition current in the WAAM process, which leads to a rapid cooling rate and results in an uneven distribution of Ti and Ni in the molten pool. Therefore, Ni and Ti do not fully integrate, with some Ti diffusing into the matrix and forming a eutectic structure. Consequently, when the deposition current is set at 35 A, a small amount of the TiNi phase is formed. In the precipitated phase (P3), the Ti content is 68.52 at%, while the Ni content is 23.47 at%. This suggests that the precipitated phase may consist of Ti_2_Ni particles.

As the electric current increases, the columnar crystal structure in the deposited layer gradually differentiates, as illustrated in Figure 7b. Figure 7f provides a localized magnification, revealing numerous small “villous” structures at the grain boundaries (P5). According to energy dispersive spectroscopy (EDS) scanning analysis, the concentrations of Ti, Ni, Cr, and Fe do not show significant differences. This phenomenon may be attributed to the increased heat input, allowing the Ti in the molten pool to fully diffuse and form intermetallic compounds with Ni, Cr, and Fe. Thus, it can be inferred that this “fluff”-like phase may consist of a eutectic structure composed of Ti, Ni, Cr, and Fe elements. At the P4 position of the grain (Figure 7f), the Ti element content is significantly higher than the Ti element content at the P2 position (Figure 7e). This may be attributed to the increased heat input associated with the higher current, which facilitates the binding of Ti atoms to Ni atoms. Consequently, the increased heat input promotes atomic diffusion and enhances the formation of compounds with other atoms. The concentrations of Ti, Cr, and Fe atoms in both the dendrite axis and interdendritic structure are similar. By correlating this with the X-ray diffraction (XRD) data, it can be inferred that Ti atoms interact with Cr, Fe, and Ni atoms to form intermetallic compounds such as Cr_2_Ti, Fe_2_Ti, and TiNi. Additionally, Nb and Mo atoms are not detected in the grains (P4). This may be attributed to the diffusion of heavy elements, such as Nb and Mo, into the interdendritic region during solidification, leading to their separation from the eutectic reaction with the remaining liquid phase between the dendrites [15].

**Figure 12:**
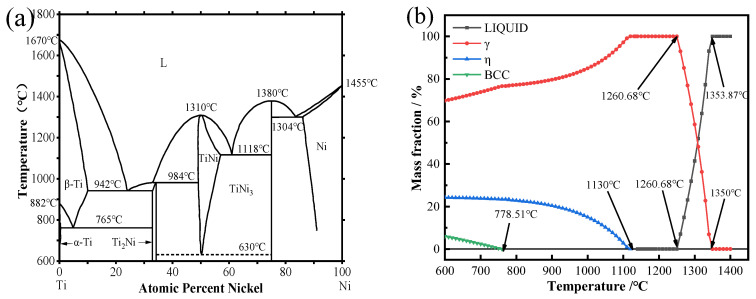
(**a**) Ti-Ni binary phase diagram [26] and (**b**) phase equilibrium fraction diagram of the composite when the deposition current is 35 A.

Figure 7c illustrates the microstructure of the sample sediment obtained with a deposition current of 45 A, while Figure 7g provides a local magnification. From Figure 7g, it is evident that numerous needle-like precipitates are present at the grain boundaries. After conducting energy dispersive spectrum (EDS) scanning analysis, it was observed that the ratio of Ni atoms to Ti atoms in the needle-like structure is approximately 3:1. According to the Ti-Ni phase diagram (Figure 12a), it can be inferred that the needle-like structure may be Ni_3_Ti, while the grain structure likely consists of γ + TiNi. In the precipitates located at position P7, the highest concentration of carbon C was detected, suggesting that the microstructure of this particular phase is MC carbide.

Figure 7d illustrates the microstructure of the sample sediment obtained with a deposition current of 50 A, while Figure 7h provides a local magnification. From Figure 7h, it can be observed that numerous acicular structures are present between the dendrites. According to the EDS scanning results, the atomic contents of Ti and Ni in the intergranular acicular structure are 22.94 at% and 45 at%, respectively. The atomic ratio of Ni to Ti is approximately 2:1. Together with the X-ray diffraction analysis results, it can be concluded that the needle structure is likely a nickel-rich TiNi phase. At the position of the precipitated phase P9, the atomic ratio of Ti to Ni is approximately 1:1, and the atomic contents of Cr and Fe are similar. It can be inferred that the precipitated phase may be a eutectic structure composed of Ti, Ni, Cr, and Fe elements, potentially forming brittle intermetallic compounds such as TiNi, Cr_2_Ti, and Fe_2_Ti. At position P11 in Figure 7h, a black precipitated phase can be observed on the dendrite axis. According to the EDS scanning results, the carbon content at P11 is higher than that of the other elements. This may be attributed to excessive heat input, leading to the partial diffusion of MC carbide into the grain during solidification. It has been reported that a small amount of MC carbide can inhibit dislocation movement and stabilize grain boundaries, thereby strengthening the material. However, an excessive amount of MC carbide can cause stress concentration and reduce the durability of the alloy [15]. When the deposition current reaches 50 A, cracks begin to appear in the sedimentary layer. This may be attributed to the stress concentration caused by the accumulation of MC carbides, as shown in Figure 2d. A detailed analysis of the crack section will be provided in the following section.

Based on the analysis above, heat input significantly affects the element content between the dendrite axis and the dendrite. Furthermore, under the same deposition current, there are considerable differences in the element content between the dendrite axis and the dendrite, indicating that the segregation mechanism is intergranular segregation. The XRD results did not detect Ti_2_Ni intermetallic compounds, possibly due to their low content. According to the Ti-Ni binary phase diagram (Figure 12a), the formation of Ti_2_Ni requires the Ti content to exceed that of Ni. Additionally, since the volume of Inconel 718 alloy in this experiment is twice that of Ti6Al4V alloy, the formation of Ti_2_Ni is unlikely. Generally, when the two alloy wires are melted in the melt pool, the internal elements should ideally diffuse evenly. However, during the arc additive manufacturing process, the extremely fast cooling rate and small deposition current lead to solidification before thorough diffusion occurs. This results in significant variations in the element content in certain areas. Although this issue improves with a gradual increase in the deposition current, cracks may still develop in the deposited layer when the current reaches a certain level due to excessive heat input.

**Figure 8:**
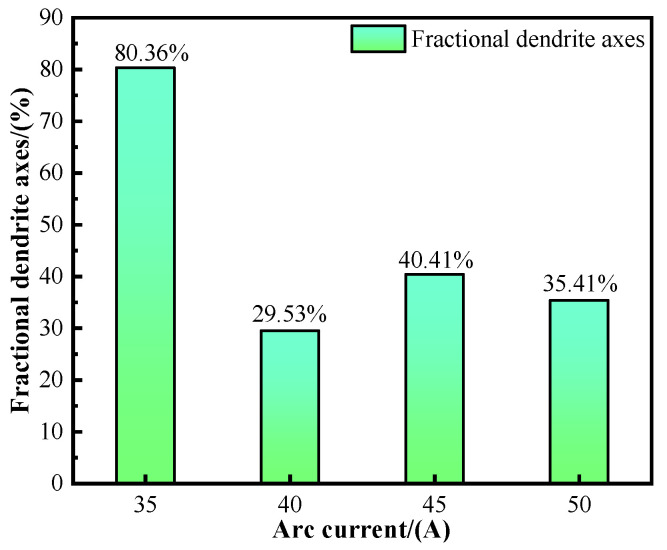
Proportion of dendrite axes.

According to SEM-EDS analysis, the Ni content along the dendrite axis is higher than the Ti content under various deposition currents, while the Ti content along the dendrite axis increases with the rising deposition current. Figure 8 illustrates the proportion of the dendrite axis. At a deposition current of 35 A, the dendrite axis proportion reaches a maximum of 80.36%. However, this proportion decreases overall as the deposition current increases. A comparison of the element content in the interdimeric and dendritic regions reveals that, with an increasing deposition current, the alloying element concentrations in these two areas become more similar, indicating a trend toward a more uniform phase distribution.

### 3.5. Cracks

**Figure 9:**
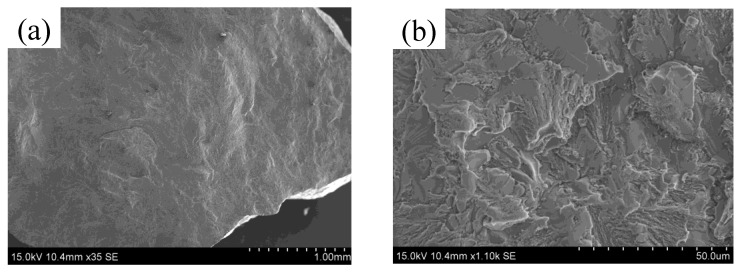

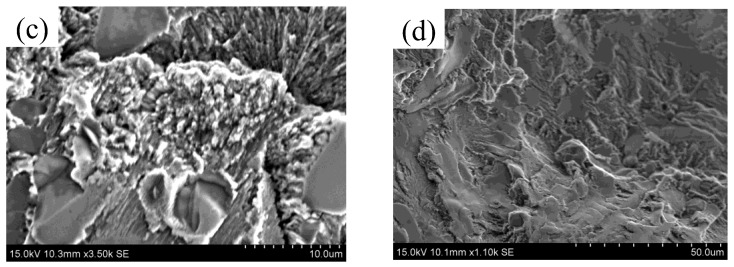
SEM images at a deposition current of 50 amps: (**a**) macroscopic profile of some cracks, (**b**–**d**) high magnification SEM images.

Titanium alloys are important structural materials, while nickel-based alloys are considered superalloys. Due to their high thermal expansion coefficients, cracks are prone to develop during deposition. In the WAAM process, no cracks were observed in the deposited layers at deposition currents below 50 A. However, at a deposition current of 50 A, cracks were detected in the deposited layer. Figure 9 illustrates the macroscopic view of the partial cracks, as well as high-rate SEM images. No cracks were found in the first layer, but the crack section in the second layer Ti6Al4V/Inconel 718 appeared disconnected, with an uneven crack morphology. High-magnification SEM images (shown in Figure 9b–d) of the crack section reveal a microfracture morphology characterized by river and cleavage patterns, indicating a cleavage fracture mechanism.

**Figure 10:**
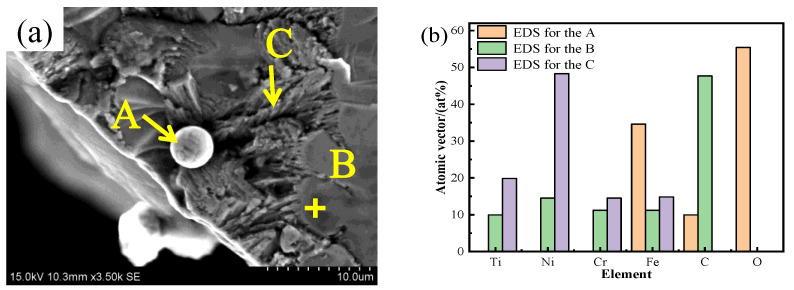

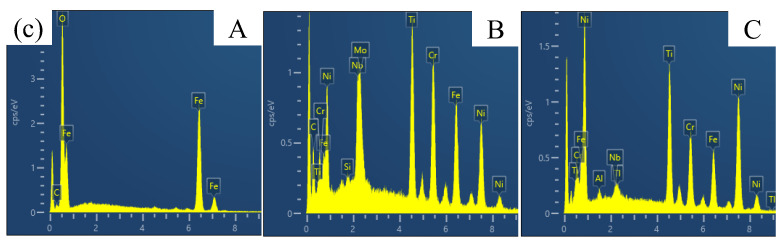
(**a**) High-magnification SEM image of the bottom of the fracture at a deposition current of 50 A. (**b**) Comparison of elemental content, (**c**) EDS-measured content map at three points in (**a**).

To investigate the phase composition of the crack section, an EDS scanning analysis of its microstructure was conducted. Figure 10 presents a high-magnification SEM image of the fracture bottom, revealing a precipitated phase A. EDS analysis indicates that the atomic ratio of oxygen to iron is approximately 1.6:1, suggesting that the precipitate is likely iron oxide. At position B, the ratio of nickel to titanium atoms is about 1:1, with the chromium and iron content being similar to that of titanium and nickel. However, the carbon content is relatively high, leading to the speculation that the main phase at position B is MC carbide. Previous studies have shown that small amounts of MC carbide can impede dislocation movement and stabilize grain boundaries, thus providing strengthening; however, excessive MC can result in stress concentration, reducing the alloy’s durability and contributing to crack formation [15]. At position C, the nickel atom content is highest, with titanium, chromium, and iron content being comparable. This leads to the speculation that the phase at this position may be a eutectic structure of brittle intermetallic compounds formed by Ni, Ti, Cr, and Fe.

As can be seen from Figure 9, the cross-section is distributed with a large number of river patterns and a “step” shape. According to XRD results and EDS scanning analysis, brittle intermetallic compounds of Ti-Fe, Ti-Ni, and Ti-Cr series are distributed in the sedimentary layer prepared by WAAM [28,29]. When the deposition current is below 50 A, the heat input is minimal, and the yield strength of the Ti6Al4V and Inconel 718 alloys exceeds the thermal stress in the deposited layer, resulting in no crack formation. However, when the deposition current exceeds 50 A, the stress concentration in the deposited layer surpasses the yield strength of the alloy, leading to crack formation.

### 3.6. Mechanical Properties

**Figure 11:**
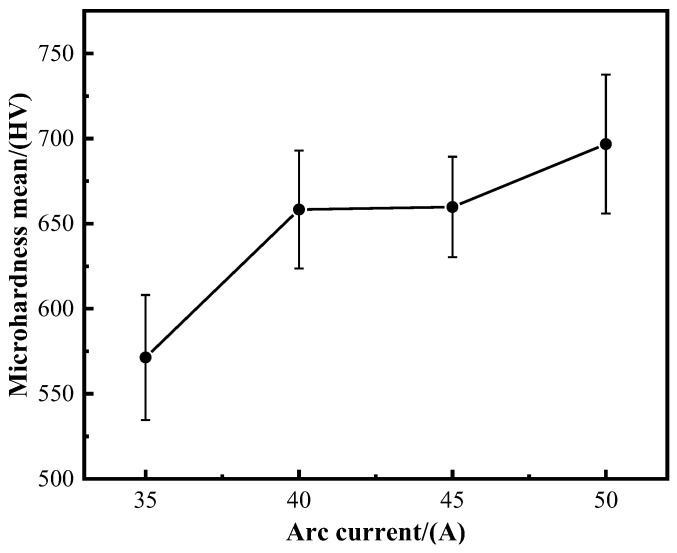
Microhardness characteristics of samples with different deposition currents.

Figure 11 presents the microhardness characteristics of samples subjected to various deposition currents, as measured by a microhardness tester. At a deposition current of 35 A, the average microhardness is 550.14 HV. This value increases to 658.27 HV at 40 A and reaches 659.79 HV at both 45 A and 50 A. The maximum recorded average microhardness is 723.50 HV. Previous studies report that the microhardness of Inconel 718 is approximately 250 HV [30]. The results indicate a significant enhancement in the microhardness of the Ti6Al4V composite, which increases progressively with higher deposition currents. 

As the deposition current increases, the heat input also rises, leading to changes in the secondary dendrite spacing, grain size, and morphology of the deposited layer. A higher deposition current results in smaller grains within the deposited layer, which increases the relative area of the grain boundaries and hinders dislocation movement. This obstruction contributes to an increase in yield strength, subsequently affecting the microhardness of the sample. Previous analysis shows that at a deposition current of 35 A, the deposited layer primarily consists of columnar crystals. These crystals are predominantly nickel-based alloys with a small amount of Ti distributed within them through diffusion. The incorporation of Ti elements enhances the microhardness of the material.

At a deposition current of 40 A, the microstructure of the deposited layer is primarily composed of intermetallic compounds such as TiNi, Cr_2_Ti, and Fe_2_Ti. Both Cr_2_Ti and Fe_2_Ti are hard and brittle intermetallic phases that contribute to the overall hardness of the material. When the deposition current increases to 45 A, there is little change in the microhardness compared to 40 A. This stability may be attributed to the microstructure along the dendrite axis, which is predominantly a nickel-based alloy, with a small distribution of Ti. The content of hard and brittle intermetallic compounds between the dendrite axis and the dendrites is minimal; however, MC carbides are located near the grain boundaries. These carbides hinder dislocation movement, thereby enhancing the material’s hardness and potentially explaining the limited change in microhardness.

When the deposition current is 50 A, the content of hard brittle intermetallic compounds on the dendrites axis and the dendrites is relatively higher, and it can be seen from Figure 7 that MC carbides are also distributed on the dendrites axis. It has been reported that a small amount of MC carbide provides strengthening by preventing dislocation movement and stabilizing grain boundaries [15], so the corresponding hardness is relatively increased. When the current is increased to 40 A, the microhardness is increased by 19.65% compared to the current of 35 A. When the deposition current increases to 50 A, the microhardness increases by 31.51% compared with that when the deposition current is 35 A.

## 4. Discussion

### 4.1. Solidification of Composite Materials Under Different Deposition Currents

In the composite materials prepared by Ti6Al4V and Inconel 718 alloys, the intermetallic compounds are mainly dominated by the Ti-Ni binary system. Figure 12 shows the Ti-Ni binary phase diagram [31]. According to the analysis of the Ti-Ni binary phase diagram, when the content of Ti and Ni atoms is close to 1:2, the eutectic reaction will occur, and new phase TiNi and Ni_3_Ti metal compounds will be formed in the composite material. However, Inconel 718 alloy contains more elements, and in the WAAM process, elements other than Ni in Inconel 718 alloy will still combine with Ti elements to form Ti-Fe phase and Ti-Cr phase. It has been reported that Fe_2_Ti metal compounds are described as the C14_LAVES phase [32] in crystallography, while Cr_2_Ti metal compounds are also described as the Laves phase [33].

The theoretical basis of the thermodynamic calculation is the CALPHAD method (computational phase diagram) [34]. When the Gibbs energy of the whole system is at its minimum, the system is in equilibrium. In some regions, the equilibrium state may be a mixture of various phases or simply a stable single phase. In a multicomponent system, the Gibbs energy of the phase can generally be expressed by the following equation:

(3) $$G_{m}=\sum_{i}x_{i}G_{i}^{o}+RT\sum_{i}x_{i}lnx_{i}+\sum_{i}\sum_{j}>1x_{i}x_{j}\sum_{V}\Omega_{V}{\left(x_{i}-x_{y}\right)}^{2}$$

where $G_{m}$ is the Gibbs energy of the phase, $\sum_{i}x_{i}G_{i}^{o}$ is the Gibbs energy of pure components, $RT\sum_{i}x_{i}lnx_{i}$ is the ideal entropy, $\sum_{i}\sum_{j}>1x_{i}x_{j}\sum_{V}\Omega_{V}{\left(x_{i}-x_{y}\right)}^{2}$ is a paired interaction term with interaction parameters, $x_{i},x_{y}$ are the molar fraction of the different alloy components, T is temperature, and R is a gas constant. $\Omega_{V}$ is the interaction coefficient that depends on the value of V. When the value of V provides values of 0 and 1, it corresponds to the normal solution model. In practice, the value usually does not exceed 2 [35].

The equilibrium phase diagram of multicomponent alloy and the fraction of phase equilibrium at each temperature can be calculated by the thermodynamic calculation principle [36]. Although there is a deviation between the non-equilibrium heating and cooling process and the theoretical temperature in the equilibrium state, the relative magnitude relationship between each temperature is unchanged, so the theoretical temperature in the equilibrium state can be used to analyze the solidification and precipitation of each phase in the cooling process.

In this work, EDS analysis was carried out on the dendrite axes and interdendrites in the composite materials under different deposition currents, the average alloying elements contents were obtained, and the solid–liquids theoretical temperature was calculated to obtain the precipitation temperature of the corresponding components. The results show that no Laves phase is found in the composite prepared when the deposition current is 35 A, that is, no Laves phase is precipitated during the cooling process. During the cooling process, γ, η, and BCC phases were precipitated from the dendritic axis. It has been reported that the η phase is an intermetallic compound phase based on the Ni_3_Ti component [37]. The structure of BCC is body-centered cubic, and the structure of the TiNi phase is reported to be body-centered cubic [31]. 

Figure 12b shows the mass fraction and temperature curves of precipitation and liquefaction of γ phase, η phase, and BCC phase calculated under the equilibrium state when the deposition current is 35 A. It can be seen that the liquid phase temperature of the dendrite axis is high, so the dendrite axis solidifies first in the cooling process, and the dendrite axis will precipitate the γ phase, η phase, and BCC phase in turn during the cooling process. The solidification process involves the redistribution of solute elements. Considering that the precipitated phase is mainly related to Ni and Ti, it can be seen from the analysis of the Ti-Ni binary phase diagram in Figure 12a that the precipitated phases on the dendrite axis are γ-Ni, Ni_3_Ti, and a small part of TiNi. When the temperature is higher than 1353.87 °C, all liquefaction is the liquid phase. When the temperature is lower than 1350 °C, the γ phase begins to precipitate. At this temperature, the dendrite axis begins to solidify, because the high-temperature existence time is very short, so the molten pool in the deposition process is basically in the solid–liquid mixing stage; in the cooling process, the dendrite and dendrite axis will form a liquid film, the liquid metal formation of heat absorption and surface tension mechanical action, resulting in the growth of the grain being limited and hindered, and then the austenite grain boundary belongs to the chemical grain boundary [38,39].

**Figure 13:**
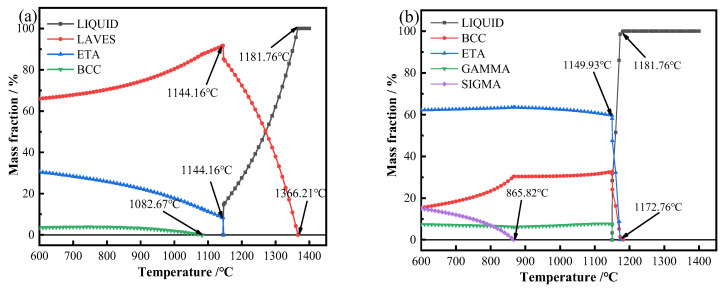
Equilibrium fraction of the composite at a deposition current of 40 A: (**a**) interdendritic and (**b**) dendrite axis.

Figure 13 shows the equilibrium fraction diagram of the composite material when the deposition current is 40 A, and the calculated results show that the temperature of the dendrite axis (Figure 7f-P4) and interdendrite (Figure 7f-P5) for complete liquefaction to liquid phase is 1172.76 °C. However, the precipitation temperature of the interdendritic Laves phase is 1366.21 °C, indicating that the interdendritic Laves phase is the first to precipitate during the cooling process, and the temperature at which its content reaches the highest point is 1144.16 °C. When the temperature is reduced to around 1181.76 °C, the Ni_3_Ti phase and TiNi phase on the dendritic axis begin to precipitate. During the precipitation of the Laves phase between dendrites, the Ni_3_Ti phase and TiNi phase on the dendritic axis also precipitate successively, and the content of the Ni_3_Ti phase hardly changes with the decrease in temperature. It is worthy of note that when the temperature is reduced to 865.82 °C, there is a discernible decrease in the TiNi phase content, accompanied by the onset of σ phase precipitation. The Laves phase has a high melting temperature, so the dendrites solidify first, which forms a liquid film with the dendrite axis, which retains the dendritic structure state, indicating that the dendrites are dispersed during the whole cooling process, as shown in Figure 7b,f.

**Figure 14:**
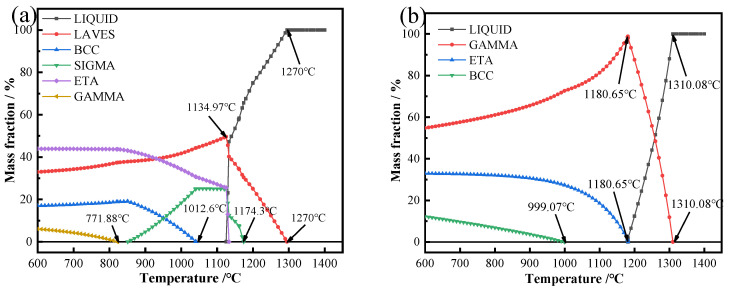
The equilibrium fraction of the composite at a deposition current of 45 A: (**a**) interdendritic and (**b**) dendrite axis.

Figure 14 shows the equilibrium fraction diagram of the composite material when the deposition current is 45 A. The calculation results show that the temperature at which all the dendrites are converted to the liquid phase is 1310.08 °C, and the temperature at which all the interdendrites are converted to the liquid phase is 1270 °C. It can be seen that during the cooling process, the γ-Ni phase solidifies first in the dendrite axis, and the temperature of the highest precipitation content of the γ-Ni phase is 1180.65 °C. The precipitation temperature of the Laves phase between dendrites is 1269.52 °C, which is similar to that of the γ-Ni phase. In the WAAM process, the high-temperature residence time is short, and the cooling rate is extremely fast, so during the cooling process, the γ-Ni phase and Laves phase precipitate almost simultaneously. With the decrease in temperature, the Ni_3_Ti phase begins to precipitate on the dendrite axis. When the temperature further decreases, the γ-Ni content on the dendrite axis decreases, while the TiNi phase content increases. The main Ni_3_Ti phase is precipitated between dendrites. When the temperature drops gradually, the content of the Laves phase decreases, and the content of the Ni_3_Ti phase increases.

**Figure 15:**
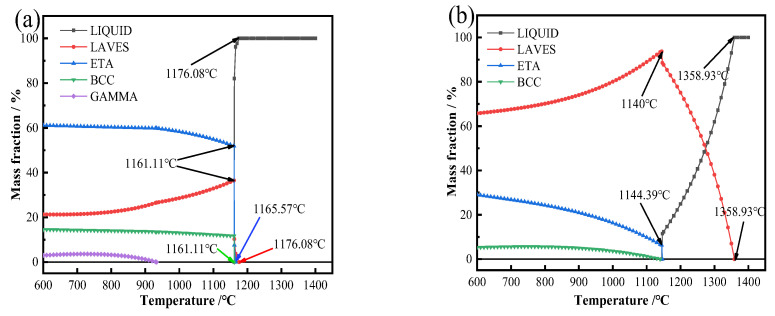
Equilibrium fraction of the composite with a deposition current of 50 A: (**a**) interdendritic and (**b**) dendrite axis.

Figure 15 presents the equilibrium fraction diagram of the composite at a deposition current of 50 A. The calculated results indicate that the precipitated phases include the Laves phase, Ni_3_Ti phase, and TiNi phase, occurring both along the dendrite axes and in the interdendritic regions. The precipitation content of the Laves phase on the dendrite axis peaks at 1140 °C, subsequently decreasing as the temperature lowers. Conversely, the contents of the Ni_3_Ti phase and TiNi phase gradually increase and approach a state of equilibrium.

The precipitation temperatures for the Laves, TiNi, and Ni_3_Ti phases are approximately 1161.11 °C. As the temperature decreases, the content of the Laves phase tends to decrease and eventually reaches equilibrium, while the content of the Ni_3_Ti phase slowly increases, also trending toward equilibrium. Throughout the solidification process from the liquid to solid state, the Laves phase on the dendrite axis preferentially precipitates, forming a liquid film between the dendrites, which helps maintain the dendritic structure.

### 4.2. Crack Analysis

The size of the welding heat input significantly influences the stress levels in the workpiece; generally, higher heat input leads to increased stress [40]. In this study, cracks occurred in the prepared composites at a deposition current of 50 A. As the deposition current increases, the high-temperature residence time is prolonged. SEM-EDS analysis of the composite material reveals the presence of MC carbides along the dendrite axis, indicating that these carbides precipitate during the solidification of liquid metal. The presence of MC carbides contributes to stress concentration in the workpiece, which can deteriorate its mechanical properties. Additionally, the presence of Laves phases also impacts the mechanical performance of the workpiece.

Microstructural analysis of the composites under different deposition currents reveals that at a deposition current of 50 A, there is a significant presence of Laves phases in both the interdendritic regions and along the dendrite axes. The presence of these Laves phases predisposes the workpiece to liquefaction cracking, adversely affecting its mechanical properties. Furthermore, SEM and EDS analyses of the fractured sections indicate that the failure mode is a cleave fracture, with a substantial presence of secondary phases detected at the fracture site. Solidification precipitation analysis suggests that these secondary phases primarily consist of the Laves phase, Ni_3_Ti, and TiNi. XRD analysis also indicates the presence of Fe_2_Ti and Cr_2_Ti in the composite. It has been documented that the Fe_2_Ti, Ni_3_Ti, and Cr_2_Ti phases are brittle intermetallic compounds [41,42]. The combined influence of the Laves phase and these secondary phases contributes to the crack formation in the composite.

### 4.3. Hardness Analysis

The microhardness of the composite increases with the increase in the deposition current. The secondary phase precipitation will also affect the microhardness of the composite. When the deposition current is small, the secondary phases such as Ti-Cr and Ti-Fe in the composite are less precipitated. When the deposition current increases, more secondary phases are generated, which increases the microhardness of the composite. In addition, the dendrites of the material are often filled with crystals with high solute content, resulting in segregation, which will affect the mechanical properties of the alloy, and the smaller the dendrite spacing, the more favorable the alloy properties [43]. The secondary dendrite spacing of composites produced under different deposition currents shows some variability. As illustrated in Figure 6, the secondary dendrite spacing initially increases with the deposition current and then decreases at higher currents. Concurrently, the microhardness of the composites also increases with the deposition current. At deposition currents of 35 A and 40 A, the secondary dendrite spacing increases, and the microhardness of the composites rises as well. This increase in microhardness at 40 A is attributed to a greater amount of secondary precipitates forming within the material, which enhances its properties. For deposition currents of 40 A and 45 A, the secondary dendrite spacing remains relatively constant, leading to similar microhardness values. However, when the deposition current reaches 50 A, the secondary dendrite spacing is reduced, and the precipitation of secondary phases is significantly higher, resulting in the highest microhardness observed.

## 5. Conclusions

1. The Ti6Al4V/Inconel 718 composite material was successfully fabricated using Ti6Al4V and Inconel 718 welding wires as raw materials, employing the double-wire Wire and Arc Additive Manufacturing (WAAM) deposition method at various deposition currents. However, it was observed that an increase in the deposition current correlated with the emergence of cracks within the composite material.
2. As the deposition current increases, the secondary dendrite spacing in the deposition samples first increases and then decreases. At a deposition current of 35 A, the secondary dendrite spacing measures 5.16 μm, while at 50 A, it reduces to 3.96 μm. Notably, the samples prepared at a deposition current of 35 A exhibit good morphology, with no visible cracks or defects.
3. At a deposition current of 35 A, the microstructure primarily consists of a nickel-based alloy, with Ti elements dispersed along the dendrite axis through diffusion. At deposition currents of 40 A and 45 A, the contents of titanium, nickel, chromium, and iron between the dendrite axis and the dendrites show minimal variation. However, at 50 A, TiNi, Cr_2_Ti, and Fe_2_Ti are predominantly deposited on the dendrite axis, while MC carbide is also found there. Additionally, Ni_3_Ti is mainly deposited between the dendrites.
4. At a deposition current of 50 A, cracks appear in the prepared sample, exhibiting a cleavage fracture. The excessive heat input leads to stress concentration in the deposited layer, surpassing the yield strength of the alloy and resulting in a cracked cross-section.
5. The hardness data indicate that the microhardness of the sample increases with the current. Specifically, when the current rises to 50 A, the microhardness shows a 31.51% increase compared to the value at 35 A.

## Figures and Tables

**Table 1:** Chemical composition of Ti6Al4V welding wire (wt%).

Al	V	Fe	C	N	H	O	Ti
5.9	3.97	0.05	0.10	0.05	0.01	0.10	Bal.

**Table 2:** Chemical composition of Inconel 718 wire (wt%).

Ni	Cr	Nb	Mo	Ti	Al	Si	Fe
53.61	18.76	4.86	3.12	1.05	0.49	0.18	Bal.

**Table 3:** Deposition parameters.

Parameter	Value
Wire feed rate (Ti6Al4V)	400 mm/min
Wire feed rate (Inconel 718)	800 mm/min
Gas flow	15 L/min
Plate speed	0.9 mm/s

**Table 4:** EDS point scanning results of each micro-area (at.%).

Area	Ti	Ni	Cr	Fe	Nb	Mo	C
P1	4.05	17.78	12.79	10.54	6.55	2.52	44.45
P2	6.1	48.14	22.89	19.64	-	-	-
P3	68.52	23.47	-	-	-	-	-
P4	17.15	43.48	21.31	16.02	-	-	-
P5	20.22	27.59	20.96	20.86	6.10	3.16	-
P6	12.87	33.63	21.89	19.56	6.18	3.66	-
P7	5.23	11.72	12.91	11.01	4.57	2.13	51.63
P8	8.20	44.65	23.81	21.59	23.81	-	-
P9	22.48	26.71	21.91	21.17	4.12	2.59	-
P10	22.94	45	16.96	15.10	-	-	-
P11	12.60	17.08	14.59	13.68	3.71	2.10	35.58

## Data Availability

The original contributions presented in the study are included in the article, further inquiries can be directed to the corresponding authors.
